# Diagnostic Accuracy of Transvaginal Sonography in the Detection of Uterine Abnormalities in Infertile Women

**DOI:** 10.5812/iranjradiol.8063

**Published:** 2012-09-17

**Authors:** Maryam Niknejadi, Hadieh Haghighi, Firoozeh Ahmadi, Fatemeh Niknejad, Mohammad Chehrazi, Ahmad Vosough, Deena Moenian

**Affiliations:** 1 Department of Reproductive Imaging, Reproductive Biomedicine Research Center, Royan Institute for Reproductive Biomedicine, Tehran, Iran; 2 Department of Reproductive Imaging, Reproductive Biomedicine Research Center, Royan Institute for Reproductive Biomedicine, Iranian Academic Center for Education, Culture & Research (ACECR), Tehran, Iran

**Keywords:** Ultrasonography, Diagnosis, Uterus

## Abstract

**Background:**

Accurate diagnosis of uterine abnormalities has become a core part of the fertility work-up. A variety of modalities can be used for the diagnosis of uterine abnormalities.

**Objectives:**

This study was designed to assess the diagnostic accuracy of transvaginal ultrasonography (TVS) in uterine pathologies of infertile patients using hysteroscopy as the gold standard.

**Patients and Methods:**

This was a cross-sectional study carried out in the Department of Reproductive Imaging at Royan Institute from October 2007 to October 2008. In this study, the medical documents of 719 infertile women who were investigated with transvaginal ultrasound (TVS) and then hysteroscopy were reviewed. All women underwent hysteroscopy in the same cycle time after TVS. Seventy-six out of 719 patients were excluded from the study and 643 patients were studied. TVS was performed in the follicular phase after cessation of bleeding. Sensitivity, specificity, positive predictive value (PPV) and negative predictive value (NPV) were calculated for TVS. Hysteroscopy served as the gold standard.

**Results:**

The overall sensitivity, specificity, positive and negative predictive values for TVS in the diagnosis of uterine abnormality was 79%, 82%, 84% and 71%, respectively. The sensitivity and PPV of TVS in detection of polyp were 88.3% and 81.6%, respectively. These indices were 89.2% and 92.5%, respectively for fibroma, 67% and 98.3%, respectively for subseptated uterus and 90.9% and 100%, respectively for septated uterus. Adhesion and unicornuated uterus have the lowest sensitivity with a sensitivity of 35% and PPV of 57.1%.

**Conclusion:**

TVS is a cost-effective and non-invasive method for diagnosis of intrauterine lesions such as polyps, submucosal fibroids and septum. It is a valuable adjunctive to hysteroscopy with high accuracy for identification and characterization of intrauterine abnormalities. This may lead to a more precise surgery plan and performance.

## 1. Background

Diagnosis of congenital anomalies and acquired diseases of the uterus have long been obstacles to successful treatment of infertility. Uterine factors are responsible for 2-3% of infertility cases and intra-uterine lesions are much more common in infertile women (40-50%) (1). Structural pathologies in the uterine cavity such as congenital mullerian anomalies and intrauterine lesions can affect endometrial receptivity, resulting in implantation failure that manifests as recurrent pregnancy loss or infertility (2). Since the results of TVS and hysterosonography and hysterosalpingography are mostly in agreement with each other, this modality has recently become the only mandatory step in the initial investigation of uterine abnormalities before resorting to invasive procedures such as hysteroscopy (3, 4, 5). TVS is readily available and cost effective and non-invasive, therefore it is universally preferred as the initial diagnostic procedure for evaluating uterine structural pathologies. It is also well-known as a valuable adjunctive to hysteroscopy and laparoscopy for identification and characterization of intrauterine abnormalities. This may lead to a more precise surgery plan and biopsy performance (6). A similar study was previously published at Royan Institute when the diagnostic accuracy of transvaginal sonogarphy in infertile patients with endometrial polyp was evaluated (7). In that study, we had a conclusive look at endometrial polyps because of the high frequency of this abnormality between all intrauterine abnormalities (8) and its importance in infertility. The increased awareness of radiologists towards intra-uterine lesions can lead to a more accurate treatment.

## 2. Objectives

In the recent study we aim to evaluate the diagnostic efficacy of TVS in the detection of all uterine abnormalities.Sensitivity, specificity, positive predictive value (PPV) and negative predictive value (NPV) were calculated for TVS considering hysteroscopy as the gold standard.

## 3. Patients and Methods

### 3.1. Patient Selection

This study was performed at Royan Institute (Infertility Clinic & Reproductive Biomedicine Research) and approved by Royan Research Center Ethics Committee. The patients were 20 to 45-year-old healthy infertile women with a history of primary or secondary infertility of more than 1 year. Exclusion criteria were as follows: all patients with no accurate visualization of the endometrium due to sonographic evaluation in improper time, heterogenic or echogenic endometrium because of bleeding. The indications for hysteroscopy were respected failed IVF or failed IUI, observed focal endometrial pathology such as polyp or submucosal myoma, irregular endometrium and synechiae, mullerian anomalies observed in hysterosalpingography or TVS.

### 3.2. Sonographic Examination

TVS was performed in the follicular phase of the cycle (days 5-13) after cessation of bleeding and before diagnostic hysteroscopy using an Aloka α-10-color Doppler with a transvaginal 6 MHz probe. TVS and hysteroscopy were both performed in the same cycle. All sonographic examinations were done by an expert radiologist with 10 years of experience. The endometrial cavity was inspected in two perpendicular plane sagittal and transverse views. Irregularities, thickness, echo pattern and any distortion of the endometrium were noted. Uterine cavity abnormalities including polyp lesions, uterine fibroids, uterine congenital anomalies such as septum, adhesion and endometrial hyperplasia were investigated. A polyp is a round or oval localized echogenic lesion located in the endometrial cavity with intact endometrial-myometrial junction. Submucosal fibroma is a benign tumor that originates from the smooth muscle layer and the accompanying connective tissue of the uterus and is seen in sonography as a mixed or hypoechoic mass lesion that originates from the myometrium and interrupts the endometrial stripe. Other types of fibroma such as intramural and subserosal types were excluded from this study because diagnosis of these pathologies is done by laparoscopy and MRI. Septum is a form of congenital malformation that partitions the uterine cavity by a longitudinal short or long wall where the outside of the uterus has a normal typical concave shape. Other kinds of congenital anomalies such as unicornuated uterus, bicornuated uterus and didelphys are also evaluated. The documentation of congenital uterine anomalies was based on classification of the American Society for Reproductive Medicine. The best view for detection of any congenital anomaly is the transverse plane in two dimensional sonography and the coronal view in three dimensional sonography. Adhesion is a fibrous band which separates the endometrial cavity and causes an abnormal adhesion which is detected as an irregular endometrial line in sonography. Hyperplasia is a condition of excessive proliferation of the cells of the endometrium which presents as thickening of the endometrium detected on sonography.

### 3.3. Hysteroscopic Examination

**Figure 1:**
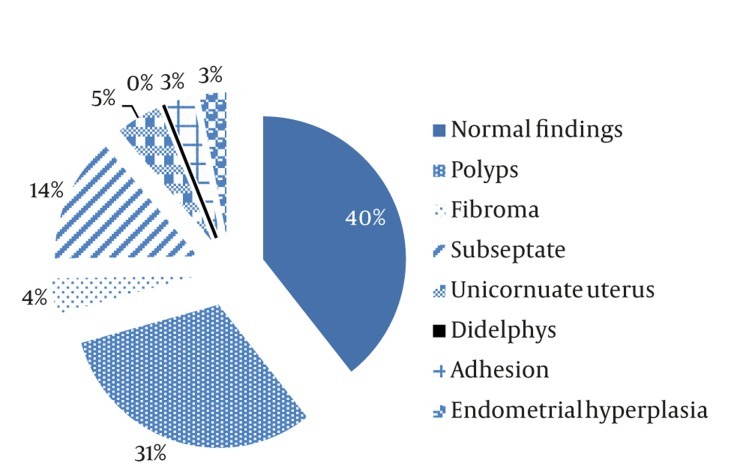
Percentage of uterine pathologies diagnosed by hysteroscopy

Hysteroscopy was performed under general anesthesia using a Storz 4mm hysteroscope (Karl. Storz – GmbH and Co. Tuttlingen, Germany) by an expert gynecologist. Distention of the cavity was obtained using a glycine serum. The number, size and location of the endometrial polyps or myoma were investigated. All women with significant clinical abnormalities which were detected by hysteroscopy underwent operative hysteroscopy at once and the specimens obtained were sent for histological examination. The percentage of uterine pathologies diagnosed by hysteroscopy is illustrated in a pie chart in Figure 1. In this study, the gynecologist was not blind to the ultrasonography results.

### 3.4. Statistical Methods

The sensitivity, specificity, positive and negative predictive values of TVS and also the positive and negative likelihood ratio with confidence intervals were calculated with hysteroscopic findings used as the gold standard. Data were analyzed using Stata version 11 (Stata Corp., College Station, TX).

## 4. Results

A total of 643patients were evaluated with both sonography and hysteroscopy. In the sight of hysteroscopy, 253 cases were detected as normal (39%) and 394 cases were pathologic, which included 197 polypoid lesions (50%), 33 long uterine septa (8%), 91 short uterine septa (23%), 28 uterine fibroids (7%), 21 adhesions (5%) and 16 endometrial hyperplasia (4%). Sonography and hysteroscopy results were in agreement in 350 pathologic cases (88.1%). However, sonography was not able to diagnose some pathologies such as 23 polypoid lesions, 30 subseptated, three long uterine septa, 14 adhesions, seven endometrial hyperplasia and three unicornuate uteruses (Table 1).

**Table 1:** Total Abnormal Findings by TVS and Hysteroscopy in 643 Patients

	**TVS a**	**Hysteroscopy**
Normal Findings	293	253
Polyps	213	197
Fibroma	25	28
Subseptated Uterus	61	91
Septated Uterus	30	33
Adhesion	7	21
Endometrial Hyperplasia	9	16
Unicornuate Uterus	4	7
Didelphys	1	1
Total Abnormal Findings	350	394
Total	643	647

^a^Abbreviation: TVS, transvaginal ultrasonography

### 4.1. Polyp Lesions

Of the total 643 accomplished hysteroscopies, polypoid lesions were observed in 197 patients (30%). In this respect, hysteroscopy was in agreement with sonography in 174 patients (88.3%), while sonography failed to detect 23 of the 197 cases (11.7%). Of the total 197 diagnosed polyps, 132 cases were sent to the pathology lab and 110 cases (83%) were confirmed; moreover, 21 proliferative endometriums and one endometrial hyperplasia were diagnosed by pathology. The residual 65 cases were not sent to the pathology lab or pathologic results were not referred to the clinic by patients. Out of the 39 cases in which sonography had detected polyps within the endometrium and hysteroscopy had not, 14 patients underwent Dillation and Curettage operation and in three patients (21%), polypoid lesions were observed, so we suggest D & C diagnosis operation in similar cases (Table 2). In the false negative group where sonography had failed to diagnose polyps, 19 patients had a pathology report as follows: endometrial polyp (9/19) and proliferative endometrium (10/19). Thus, in 10 cases a misdiagnosis had occurred by hysteroscopy.

**Table 2:** Various Diagnostic Indices of TVS Compared with Hysteroscopy in Diagnosing Abnormal Uterine Findings

**diagnostic Indices**	**sensitivity (% 95 cI)**	**specificity (% 95 cI)**	**PPV a (% 95 cI)**	**NPV a (% 95 cI)**	**Positive likelihood ratio (% 95 cI)**	**Negative likelihood ratio (% 95 cI)**
Uterine Abnormalities						
Polyps	88.3 ( 82.8-92.3)	91.2 (88.1-93.6)	81.6 (75.6-86.5 )	94.6 ( 91.9-96.5)	10.1 (7.4-13.6)	0.12 (0.08-0.18)
Fibroma	89.2 ( 70.6-97.2)	99.6 (98.7-99.9)	92.5 ( 74.2-98.7)	99.5 ( 98.4-99.8)	274 (68.4-1101.8)	0.10 ( 0.03-0.31 )
Subseptated Uterus	67.0 (56.3-76.3)	99.8 (98.8-99.9)	98.3 ( 90.1-99.9)	94.6 ( 92.6-96.4)	370 (51.9-2636)	0.33 (0.24-0.44)
Septated Uterus	90.9 (74.5-97.6)	100 (99.0-100)	100 ( 85.8-100)	99.5 ( 98.1-99.8)	infinity	0.09 (0.03-0.26)
Adhesion	33.3 (15.5-56.9)	99.8 (98.9-99.9)	87.5 ( 46.6-99.3)	97.9 (96.2-98.7 )	207 (26.7-1610)	0.66 (0.49-0.90)
Endometrial Hyperplasia	56.2 (30.5-79.2)	99.6 (98.7-99.9)	81.8 ( 47.7-96.7)	98.8 ( 97.6-99.5)	176 (41.3-751.4)	0.43 (0.25-0.76)
Total	79	82	84	71		

^a^Abbreviation: NPV, negative predictive value; PPV, positive predictive value

### 4.2. Uterine Fibroids

Of the total 28 uterine fibroids observed in patients by hysteroscopy, sonography results were in agreement with the hysteroscopy results in 25 cases (89.3 %,) but there was disagreement between the two techniques in three cases (10.7 %) (Table 2). In this study, we only considered the submucosal fibroma that may be further investigated by hysteroscopy and other types of fibroma such as intramural and subserosal fibroma were ignored. Diagnosis of these types of fibroma should be done by MRI and laparoscopy.

### 4.3. Adhesion

Twenty-one cases of adhesion were observed by hysteroscopy, while sonography was not able to recognize only seven correct cases (33 %), but 14 cases were failed (Table 2). Among these failed cases, 85% (12/14) contained mild adhesions and in two severe adhesions sonography was able to diagnose correctly (Table 2).

### 4.4. Endometrial Hyperplasia

Hysteroscopy diagnosed 16 cases with endometrial hyperplasia, out of which in nine cases (56.2%) the result of sonography and hysteroscopy were in agreement, but disagreement between the results of these two techniques existed in seven cases. In the other two cases, the diagnosis of endometrial hyperplasia via sonography was not approved by hysteroscopy. In these nine cases of hyperplasia, which were confirmed with both sonography and hysteroscopy, the specimens were sent to the pathology laboratory and only in three cases hyperplasia was approved (Table 2).

### 4.5. Mullerian Anomalies

Of the total seven unicornuate uteri observed by hysteroscopy, in four cases, the results of sonography were similar to hysteroscopy (57.1%). Two unicornuate uteri were reported because the hysteroscope was entered into one cavity without any communication to the other cavity. In one case of didelphys uterus, both methods were in agreement. Hysteroscopy diagnosed 91 cases with short uterine septum and sonography was in agreement with the hysteroscopy result in 61 cases (67%), while the sonography results did not agree with that of hysteroscopy in 30 other cases (Table 2). Of the total 33 long uterine septa observed by hysteroscopy, in 30 cases, the results of sonography were similar with hysteroscopy (90.9%) and in three other cases, sonography failed to diagnose the anomalies due to a thin endometrium or fibromatose echo pattern of myometer that distorted the endometrium (Table 2).

## 5. Discussion

**Figure 2:**
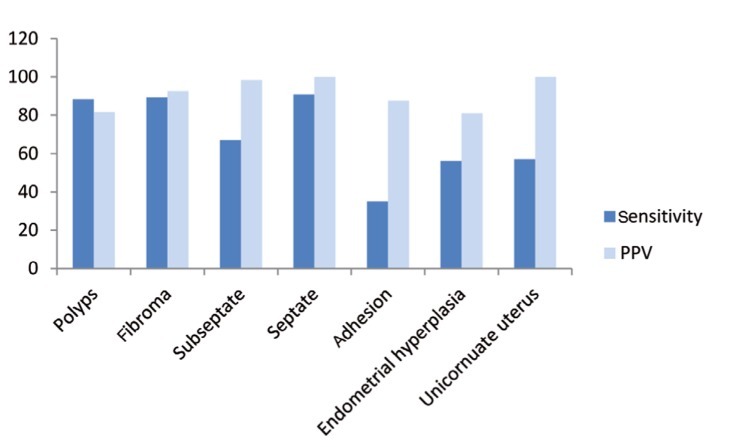
Sensitivity and PPV of TVS in different uterine pathologies during a one-year-study of infertile women

Various forms of female infertility were associated with congenital uterine anomalies and acquired uterine disease (3, 9). In general, TVS as a noninvasive and valuable diagnostic modality plays an important role in the evaluation of uterus and endometrial abnormalities (3, 10). The objective of this article was to assess the diagnostic value of TVS performed prior to routine hysteroscopy to confirm that TVS could reduce the number of diagnostic hysteroscopies commonly carried out in women with normal uterine cavities. Loverro et al. (9) and Soares et al. (11) have reported that TVS had a sensitivity and specificity of as high as (75-85%) and (90-100%), respectively for the detection of endometrial polyps. Using hysteroscopy as a gold standard, TVS showed excellent specificity (91.2%), good sensitivity (88.2%), an 81.4% PPV and a 94.6% NPV in uterine polyp detection in our setting. Likewise, the PPV of TVS for the detection of polyps in our setting was higher than that reported by Soares et al. (11). While, Fedele and colleagues reported that TVS had a misdiagnosis rate of 4.2% and was therefore less effective in distinguishing polyps than hysteroscopy (12). In cases of endometrial fibroids, TVS had a sensitivity of 89.2% and a specificity of 99.6%. These findings correlate with the result of Loverro et al. (9) in which TVS had a 90.9% sensitivity and a 100% specificity for the detection of endometrial fibroids. For the diagnosis of endometrial septum, TVS had a sensitivity of 67% and specificity of 99.8% in patients with a subseptated uterus, while the diagnostic accuracy of TVS is more significant in those patients with a long septum, which showed a sensitivity of 90.9% and specificity of 100% for endometrial long septum. In our study, TVS had 57.1% sensitivity and 100% positive predictive value for unicornuate uterus (Figure 2). Furthermore, Soares et al.(11) reported that TVS had a positive predictive value of 100% for uterine malformations detected in hysteroscopy of infertile patients. In this study, TVS failed to distinguish adhesions in 14 out of 21 patients (67%). While Fedele et al. (12) and Shalev et al. (3) reported a high accuracy of TVS in diagnosing uterine adhesions. It is recommended that in case of endometrial adhesion detected by sonography, the final diagnosis needs to be confirmed by salin infusion sonography (sonohysterography) which separates the two layers of the endometrium or by diagnostic hysteroscopy. In cases of endometrial hyperplasia, TVS had a sensitivity of 56.2% and a specificity of 99.6%. In this study, all patients were not referred for D & C and only nine cases had a pathologic report. Hyperplasia was approved in three out of nine cases and in six out of nine cases, focal hyperplasia was proved and proliferative endometrium was reported in the pathology test. Sonographic diagnosis of these three cases were correct; thus, endometrial hyperplasia cannot be diagnosed with hysteroscopy alone and the diagnosis of endometrial hyperplasia via hysteroscopy should be approved by a biopsy specimen (13). Overall, as a test for the detection of intra-uterine abnormalities, TVS had 79% sensitivity and 82% specificity, 84% positive predictive value and 71% negative predictive value in comparison with hysteroscopy. This study showed similar results to Narayan and Goswamy’s study which suggested TVS had a positive predictive value as high as 85-95% for specific uterine abnormalities detected by hysteroscopy in an infertile population (14). In this study, only histological diagnosis of resected endometrial tissues by hysteroscopy was available and obviously all patients with fibroma or mullerian duct anomalies had no histological results. In spite the fact that the most accurate diagnosis is based on pathological confirmation, the goal of this study was determining the agreement between the results of TVS and hysteroscopy which is determined by direct optic visualization which was the gold standard in the same study (15). Sonography is a safe, available and inexpensive method with multiple capacities such as 3D, 4D, Doppler studies and saline infusion (sonohysterography) which can properly diagnose uterine pathologies before hysteroscopy. Previous studies have mostly shown that sonohysterography is less invasive than hysteroscopy and in some circumstances may obviate diagnostic hysteroscopy (16-18). It may provide a specific diagnosis and enable the surgeon to proceed to operative hysteroscopy. This method is cost-effective, less complicated and less time consuming. It may be a proper alternative for diagnostic hysteroscopy saving more time and may help the surgeon perform the procedure more accurately.We conclude that TVS as a routine procedure before hysteroscopy enables the detection of the details of most localized endometrial lesion.
